# Enhancing Esthetics in Direct Dental Resin Composite: Investigating Surface Roughness and Color Stability

**DOI:** 10.3390/jfb15080208

**Published:** 2024-07-25

**Authors:** Adrian Ioan Hajdu, Ramona Dumitrescu, Octavia Balean, Dacian Virgil Lalescu, Berivan Laura Rebeca Buzatu, Vanessa Bolchis, Lucian Floare, Diana Utu, Daniela Jumanca, Atena Galuscan

**Affiliations:** 1 Translational and Experimental Clinical Research Centre in Oral Health, Department of Preventive, Community Dentistry and Oral Health, Victor Babes University of Medicine and Pharmacy, 300040 Timisoara, Romania; hajdu.adrian@umft.ro (A.I.H.); balean.octavia@umft.ro (O.B.); berivan.haj-abdo@umft.ro (B.L.R.B.); vanessa.bolchis@umft.ro (V.B.); floare.lucian@yahoo.com (L.F.); jumanca.daniela@umft.ro (D.J.); galuscan.atena@umft.ro (A.G.); 2 Clinic of Preventive, Community Dentistry and Oral Health, Victor Babes University of Medicine and Pharmacy, Eftimie Murgu Sq. no 2, 300041 Timisoara, Romania; 3 Department of Food Science, Faculty of Food Engineering, University of Life Sciences “King Mihai I” from Timisoara, 119 Calea Aradului Street, 300645 Timisoara, Romania; lalescu@usvt.ro; 4 Department of Pharmacology-Pharmacotherapy, Faculty of Pharmacy, Victor Babes University of Medicine and Pharmacy, Eftimie Murgu Sq. no 2, 300041 Timisoara, Romania; diana.utu@umft.ro

**Keywords:** dental resin composite, surface roughness, color stability, hydrophobicity, acidic beverages, contact angle

## Abstract

Dental restorations must replicate the natural appearance of teeth while ensuring biocompatibility and durability. This study evaluated the surface characteristics and color stability of three dental composites—Herculite Ultra XRV, G-ænial A’CHORD, and Omnichroma—exposed to acidic beverages (red wine, black coffee, and Coca-Cola). Sixty disk-shaped specimens were prepared, polished, and immersed in these beverages. Surface roughness was assessed using profilometry and SEM analysis, hydrophobicity via contact angle analysis, and surface charge through streaming potential measurements. Color stability was evaluated using a spectrophotometer, and the pH levels of the solutions were recorded. Results showed that Herculite Ultra XRV had the highest mean contact angle (79.46° ± 6.52), indicating superior hydrophobicity, while Omnichroma had the lowest (64.94° ± 3.08), indicating more hydrophilicity. Significant color changes were observed, especially in black coffee, with ∆E values indicating notable discoloration. The acidic pH of the solutions increased surface roughness and color changes. Statistical analyses confirmed significant increases in surface roughness and color change for all composites, with the nanohybrid resin composite showing the greatest variability. These findings highlight the need for dental restorative materials with enhanced resistance to acidic environments to improve the longevity and esthetics of dental treatments.

## 1. Introduction

The oral cavity is an intricately structured and dynamically evolving ecosystem, where the primary contributor to caries formation is the presence of polymicrobial biofilms thriving on dental hard tissues [1]. Dental caries often correlates with a disruption in the balance of oral microflora. Dental restorative materials are commonly considered artificial surfaces prone to the attachment and buildup of oral microorganisms [2]. The attachment of bacteria to tooth surfaces and dental restorative materials plays a significant role in the onset of dental caries and related periodontal diseases [3]. The incidence of adult dental caries remains alarmingly high worldwide, with nearly 100% prevalence in many countries. This underscores the urgent need for restorative treatments to manage and mitigate the extensive impact of dental caries on overall health [4,5]. The high number of reported caries cases necessitates the use of direct restorations with resin composites, which is one of the most widespread procedures and presents challenges in ensuring long-term durability, esthetics, and biocompatibility.

Presently, resin composites are the most commonly employed materials for cavity restoration owing to their capacity for direct filling, esthetic appeal, minimal toxicity, and enhanced performance [6]. Longevity stands as a paramount consideration for both patients and dentists when it comes to dental restorations. The composition and characteristics of oral solid surfaces significantly influence the pellicle coatings. This aspect becomes particularly crucial in the context of restorative materials utilized to replace lost dentin and/or enamel tissue, as they come into contact with remaining dental tissues highly susceptible to recurrent caries [7]. Clinical research consistently underscores that recurrent caries persists as the primary reason of restoration failure over time, often attributable to biofilm formation on dental restorations [1]. Recurrent caries is a widespread issue globally, imposing substantial health and financial costs [8].

Resin composite materials consist primarily of two components: the organic resin matrix and the inorganic ceramic fillers. The resin matrix comprises monomers, diluents, photo initiators, accelerators, and coupling agents [9]. In recent times, there has been a trend towards utilizing nanoparticles and nanofibers as fillers, owing to their outstanding esthetic appeal, bioactivity, and biocompatibility characteristics [10]. Several factors influence the attainment of a highly polished surface in resin composite restorations, including the filler-to-matrix ratio, filler particle size, and the techniques employed for finishing and polishing the restoration [11]. Brushing, polishing, abrasion, erosion, and microcracking, along with exposure to acidic environments, have the potential to alter the properties of composite dental restorative materials. Hydrophobicity stands out as a crucial characteristic of these materials, influencing both initial water absorption and the adhesion of oral bacteria [12]. The contact angle method provides a mean measurement for hydrophobicity, with a lower angle indicating a more hydrophilic surface. Also, studies have demonstrated that as surface roughness increases, the contact angle on a solid surface decreases [13]. Relying on a single method for surface roughness (SR) analysis may be insufficient. Comparing findings from multiple methods can yield more accurate results. SR measurements can be conducted using quantitative methods like Scanning Electron Microscopy (SEM), as well as qualitative methods and surface profile analysis using a profilometer [14]. Also, streaming potential measurements provide valuable insights into the electrokinetic properties of composite resins, shedding light on their surface charge characteristics and potential interactions with various substances.

The consumption of certain beverages can impact the esthetic and physical properties of resin composites, potentially compromising the quality of restorations [15]. This article discussing the modifications in characteristics of different types of composites immersed in wine, coffee, and Coca-Cola addresses a critical aspect of dental restorations’ longevity and esthetic maintenance. By evaluating the changes in surface properties and color stability of dental resin composites exposed to common beverages, this study provides valuable insights into the practical challenges faced in clinical dentistry. Such research aligns with the goal of informing practitioners about new materials and technologies that can improve patient outcomes and enhance the durability of dental treatments, ultimately contributing to the expansion of the dentist’s knowledge base in a scientifically rigorous manner.

This study aimed to evaluate the surface characteristics and color stability of three distinct dental composite materials used for direct restoration: Herculite Ultra XRV (Kerr, Bolzano, Italy, S.R.L.), G-ænial A’CHORD (GC Corporation, Tokyo, Japan), and Omnichroma (Tokuyama Dental, Tokyo, Japan). The surface properties were comprehensively examined. Surface roughness was assessed using profilometry and SEM analysis, while hydrophobicity was evaluated through contact angle measurements and surface charge through streaming potential measurements. Color stability was assessed using a spectrophotometer, and the pH levels of the immersion solutions were recorded. Specifically, red wine, black coffee, and Coca-Cola were chosen for their acidity. Furthermore, color parameters of the materials were analyzed, with alterations studied by immersing the surfaces in the aforementioned beverages known for their colorant properties. The null hypothesis for this study is that there will be no significant differences in the surface characteristics and color stability of the three dental composite materials after immersion in common acidic beverages (red wine, black coffee, and Coca-Cola).

## 2. Materials and Methods

In this study, three commercial restorative materials were examined: nanohybrid resin composite known for its high esthetic properties and mechanical strength (Herculite Ultra, Kerr, Bolzano, Italy, S.R.L.), micro-hybrid resin composite chosen for its ease of handling and superior polishability (G-ænial A’CHORD, GC Corporation, Tokyo, Japan), and monocolor composite renowned for its single-shade universal application, which adapts to the surrounding tooth color (Omnichroma, Tokuyama Dental, Tokyo, Japan) (see Table 1).

### 2.1. Specimen Preparation

To prepare the specimens, 60 disk-shaped samples were fabricated, 20 for each material. The sample size for the study was determined based on the objective of achieving statistically significant results while considering the practical constraints of material availability and testing equipment capacity. We utilized the effect size of 0.8 derived from preliminary studies and similar research in the field of dental materials, a power of 0.80, and an alpha level of 0.05, resulting in a minimum of 12 samples per group, but 20 samples per group were used to ensure robustness, totaling 60 samples across three material groups.

These specimens had a diameter of approximately 10 mm and a height of about 2 mm, created using a calibrated circular plexiglass mold. A clean glass slab was placed underneath the mold to provide support and ensure adequate compaction of the materials. After filling the resin composites into the mold, the surface was covered with celluloid tape to minimize the formation of an oxygen-inhibited layer. Each side of the mold was then light-cured for 40 s using a light-curing device (Bluephase G2, Ivoclar Vivadent, Mississauga, ON, Canada) with a light intensity of 1200 mW/cm^2^ at a distance of approximately 1 mm from the surface [19]. Following curing, all specimens were removed from the mold and visually inspected. The fabricated specimens were stored in an incubator in distilled water at 37 °C for 24 h, then polished, and subsequently immersed in the beverages [20,21,22]. All composite samples underwent polishing for 20 s with each Sof-Lex disk (3M ESPE Dental Products, St. Paul, MN, USA). Successive disks of varying abrasiveness including Coarse (100 μm), Medium (29 μm), Fine (14 μm), and Super Fine (8 μm) were utilized. To ensure standardization, the polishing process was carried out by a single operator using a low-speed hand device, applying uniform, dry, and intermittent pressure at 15,000 rpm for 20 s, following the method described by Gonulol and Yilmaz (2012) [23]. Each specimen received polishing with a new disk. Subsequently, after rinsing, all specimens were subjected to ultrasonic cleaning in distilled water for 5 min.

**Figure 1:**
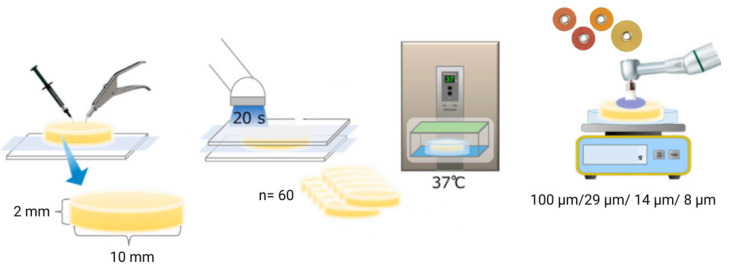
Specimen preparation.

To ensure consistency, the same operator carried out all procedures (Figure 1).

### 2.2. Contact Angle Measurements

Contact angle measurements were conducted utilizing a Drop Shape Analyzer-DSA25 (KRÜSS GmbH, Hamburg, Germany). Drop Shape Analyzer—DSA25 is a user-friendly and reliable instrument designed for measuring contact angles [24,25,26]. Whether conducting a simple wetting test or accurately measuring surface free energy (SFE), this robust device offers versatile options for analyzing wetting and adhesion on solid surfaces. The DSA25 is equipped with a high-resolution camera and a superior zoom lens, ensuring an optimal display of the drop size. This high image quality enables precise measurement of contact angles or surface tension. When combined with the ADVANCE (Version Number 1.14.1.16701) software’s intelligent image evaluation algorithm, the instrument delivers exact results through accurate drop shape analysis [27]. Dental surfaces were positioned on the sample stage, where a liquid droplet was deposited onto the material surface, and the contact angle between the droplet and the surface was recorded. The Drop Shape Analyzer-DSA25, KRÜSS GmbH, Hamburg, Germany, was used to measure the water contact angle for all composite materials. The contact angle was determined with the Double Sessile Drop method by using distilled water, and the drop volume was adjusted to 1 µL. In order to calculate the mean contact angle, 6–7 measurements were performed for each composite.

### 2.3. The Streaming Potential Measurements

The zeta potential was determined by using the equipment Particle charge detector Mutek PCD-03 (Mütek GmbH; Neckartailfingen, Germany) [28]. It is used to determine the concentration of water-soluble ionic polymer solutions and the point of zero charge for composite micro- and nanoparticles. For this measurement, 10–20 mg solid powder was suspended in 10 mL PBS solution. The measured streaming potential was read on the device display after 10 min equilibration time.

### 2.4. Roughness Analysis

Surface characterization of the dental resin composite samples was performed using a Mitutoyo SJ-201 Roughness Tester (Mitutoyo Europe GmbH, Oberndorf, Germany). Each specimen underwent three-line measurements to determine the arithmetic average roughness (Ra). The profilometer, equipped with a diamond tip of 5 μm radius, captured two-dimensional profiles at a measuring speed of 0.5 mm/s, with a cutting value of 0.8 mm and a measuring distance of 4 mm. Measurements were taken in three different areas of each material, and the arithmetic mean of these results was recorded as the peak-to-valley value (Ra) [29].

### 2.5. SEM Analysis

Scanning Electron Microscopy (SEM) was employed for the analysis of composite material samples. The SEM analysis was conducted using an Inspect scanning electron microscope under low-vacuum conditions, operating at a pressure of 60 Pa and a voltage of 30 kV, with images captured at a scale bar of 50 µm.

### 2.6. Color Measurement

Color measurements were conducted using the VITA Easyshade V (Vita Zahnfabrik, Bad Säckingen, Germany) digital spectrophotometer, which was developed for precise, fast, and reliable shade determination of natural teeth and restorations [30]. The color parameters (L*, a*, b*) were analyzed in accordance with the standards set by the Commission Internationale de l’Eclairage (CIE). Additional color parameters, namely the hue angle (°) and relative color saturation (C*), were derived from the calculations. The hue angle quantifies color on a scale typically represented on a color wheel, with 0° denoting red, 180° its complementary green, 90° yellow, and 270° its complementary blue. Relative color saturation (C*) assesses the vividness of an object’s color relative to its brightness. In this study, changes in color induced by immersion in red wine, black coffee, and Coca-Cola were assessed via colorimetry. Plates were submerged in each beverage for 20 min daily for ten consecutive days and then allowed to air-dry after removal. CIE parameters L*, a*, and b* were recorded both before and after immersion. The total color difference (∆E) was calculated using the formula:

∆E = ([∆a*]^2^ + [∆b*]^2^ + [∆L*]^2^)^1/2^

where ∆E values greater than 2.7 indicate “very distinct” changes, values between 1.2 and 2.7 indicate “distinct” changes, and values less than 1.2 indicate “non-distinct” changes [31].

### 2.7. Adherence of Red Wine, Black Coffee, and Coca-Cola

To examine the adherence of red wine, black coffee, and Coca-Cola on dental surfaces, all three types of dental composite specimens were employed. These plates were submerged in black coffee, prepared to standard infusion strength, 5 g to 150 mL boiled water (Nespresso, Nestle Romania SRL, Bucharest, Romania), red wine (Budureasca clasic, Feteasca Neagra, Dealu Mare, Romania), and Coca-Cola (The Coca-Cola Company, Atlanta, GA, USA) for a duration of 20 min daily, for 10 consecutive days [32,33,34]. Subsequently, the specimens were removed from the beverages and air-dried. The adherence of all beverages was assessed by measuring the CIE L*, a*, b* color parameters relative to the original, control specimens.

### 2.8. pH Measurement

The pH of the staining solutions was determined using a portable Milwaukee MW100 pH meter. This instrument provided accurate and reliable pH readings necessary for assessing the acidic nature of the solutions used in the study. Each solution’s pH was recorded before the immersion of the composite samples to ensure consistent conditions for evaluating the impact on the surface roughness and color stability of the resin composites. Prior to measurement, the pH meter was calibrated with standard solutions, and the electrode was rinsed with distilled water before each step.

### 2.9. Statistical Analysis

The surface roughness and color change in the esthetic restorative materials before and after immersion in each beverage were statistically analyzed using the SPSS 23.0 software (SPSS, Chicago, IL, USA). Statistical analysis was performed using one-way ANOVA and paired *t*-tests, with results considered significant at *p* < 0.05.

## 3. Results

### 3.1. Contact Angle

**Figure 2:**
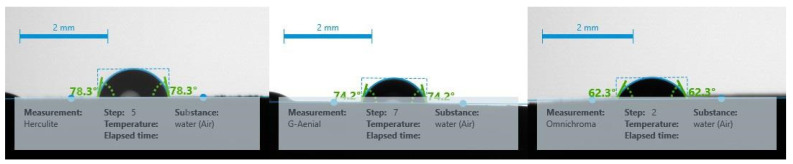
Contact angle measurements for various resin composites at different stages.

The data reveal that Herculite Ultra exhibits the highest mean contact angle at 79.46°, indicating a relatively more hydrophobic surface compared to the other composites. G-Aenial A’CHORD follows with a mean contact angle of 73.22°, while Omnichroma has the lowest mean contact angle of 64.94°, suggesting it has a more hydrophilic surface. The standard deviations indicate the variability of the measurements, with G-Aenial A’CHORD showing the least variability (2.71), and Herculite Ultra the most (6.52), as shown in Table 2 and Figure 2.

### 3.2. The Streaming Potential

**Figure 3:**
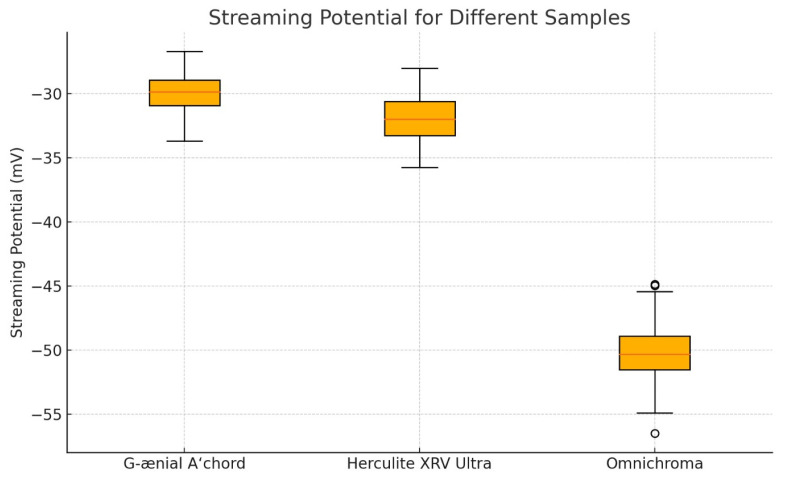
Streaming potential values for three samples measured in PBS solution (pH = 7.45).

To determine the point of zero charge (pzc), which represents the pH value where the streaming potential is zero, a pH titration of the resin composite sample was carried out (Figure 3).

**Figure 4:**
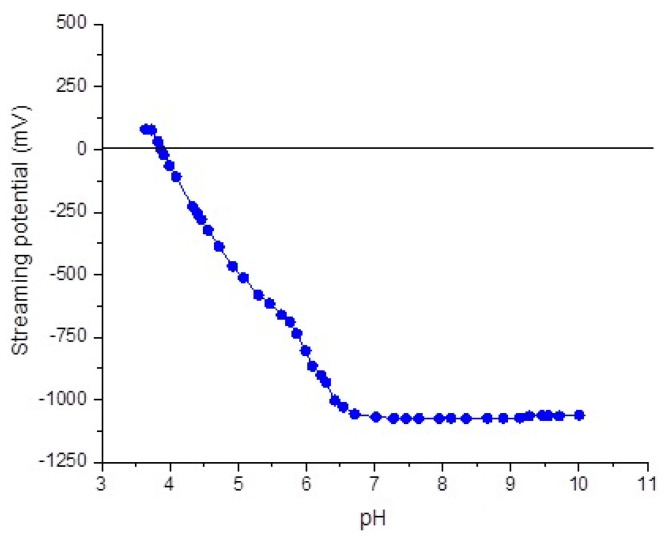
Streaming potential measurements as function of pH for solid sample.

The pH titration started from the acidic medium (a HNO_3_ solution, 0.5%, was used) toward the basic region of pH by adding NaOH aqueous solution (drop-by-drop). In this case, the pzc was situated in the acidic range of pH (3.8), demonstrating that the number of anionic ionized/ionizable groups is much higher than the positive ones (Figure 4).

### 3.3. Roughness

Statistical analyses, including descriptive statistics and paired *t*-tests, were conducted to evaluate changes in surface roughness over time. Table 3 displays the mean surface roughness values and standard deviations for the three composites at two different time points (Day 0 and Day 10). The initial surface roughness (Day 0) was generally lower than the roughness measured after 10 days of immersion in the staining solutions.

Regarding Herculite UltraXRV, the mean roughness showed varied changes after immersion in different liquids over 10 days. Initially, the surface roughness (SR) was 1.668 ± 0.45. After 10 days in coffee, the roughness decreased to 1.36 ± 0.23, indicating a smoothing effect. In contrast, immersion in red wine significantly increased the roughness to 2.28 ± 0.77, suggesting an abrasive or erosive effect. Coca-Cola immersion caused a slight increase in roughness to 1.63 ± 0.26, but this change was not as pronounced as that caused by red wine.

For G-ænial A’CHORD, the initial SR was 1.84 ± 0.33. After 10 days in coffee, the roughness increased significantly to 2.33 ± 0.62, indicating coffee’s considerable abrasive effect. Red wine immersion led to a slight decrease in roughness to 1.52 ± 0.63, whereas Coca-Cola caused a substantial increase in roughness to 3.98 ± 0.13, indicating a strong erosive effect.

Omnichroma started with an initial SR of 1.06 ± 0.09. After 10 days, coffee immersion increased the roughness to 1.53 ± 0.38, showing a moderate abrasive effect. Red wine caused a minimal increase in roughness to 1.15 ± 0.08, and Coca-Cola resulted in a slight increase to 1.21 ± 0.31, less than that caused by coffee but similar to red wine.

The statistical analyses (paired *t*-test) revealed notable changes in surface roughness for all three composites after 10 days of immersion in various solutions. For Herculite UltraXRV, coffee had a smoothing effect, red wine was erosive, and Coca-Cola caused a slight increase in roughness. For G-ænial A’CHORD, both coffee and Coca-Cola significantly increased roughness, while red wine slightly decreased it. For Omnichroma, coffee resulted in a moderate increase in roughness, red wine led to a minimal increase, and Coca-Cola caused a slight increase (see Table 3).

### 3.4. SEM Analysis

**Figure 5:**
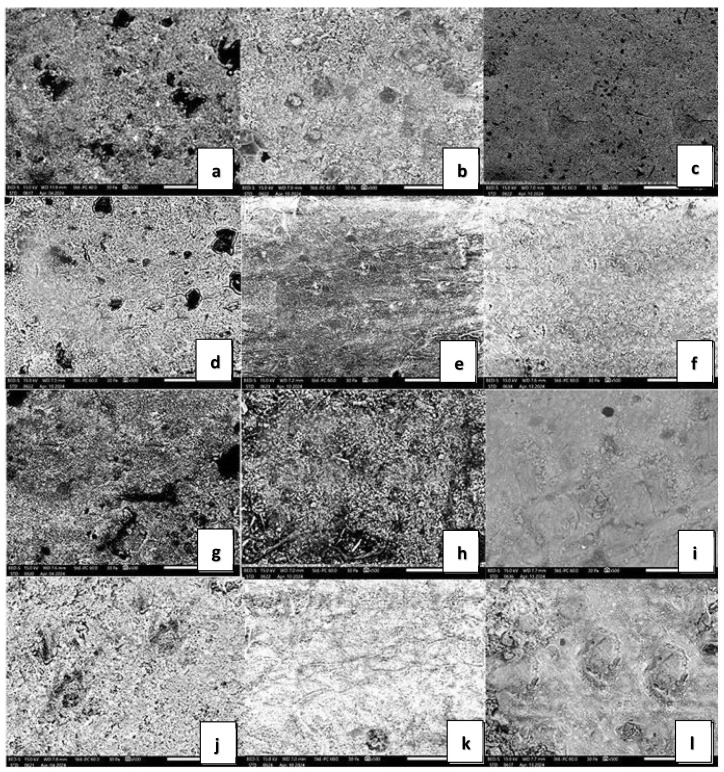
SEM analysis of surface roughness for different composite materials. Scanning Electron Microscopy of (**a**) control Herculite Ultra XRV; (**b**) control G-ænial A’CHORD; (**c**) control Omnichroma; (**d**) Herculite Ultra immersed 10 days in coffee; (**e**) G-ænial A’CHORD immersed 10 days in coffee; (**f**) Omnichroma immersed 10 days in coffee; (**g**) Herculite Ultra immersed 10 days in red wine; (**h**) G-ænial A’CHORD immersed 10 days in red wine; (**i**) Omnichroma immersed 10 days in red wine; (**j**) Herculite Ultra immersed 10 days in Coca-Cola; (**k**) G-ænial A’CHORD immersed 10 days in Coca-Cola; (**l**) Omnichroma immersed 10 days in Coca-Cola at magnification ×500.

The SEM analysis revealed distinct surface morphology changes among the three dental composites under various conditions. In the case of Herculite Ultra XRV, the control sample exhibited a relatively smooth surface with minimal defects, indicating an intact and uniform composite structure under neutral conditions. Immersion in coffee resulted in increased surface roughness with small pits and cracks, suggesting interaction between coffee components and the composite material, initiating degradation. The wine-immersed sample showed significant surface degradation, with pronounced cracks and noticeable material loss, indicating a stronger corrosive effect compared to coffee. The sample exposed to Coca-Cola demonstrated extensive surface roughness, large pits, and significant material degradation, reflecting the highly corrosive nature of Coca-Cola, likely due to its acidic content and other reactive components. For G-ænial A’CHORD, the control sample exhibited a relatively uniform surface with minor irregularities. In contrast, the coffee-immersed sample showed noticeable surface roughness and deposits, likely from organic compounds in the coffee. The wine-immersed sample displayed significant surface irregularities and a higher degree of porosity. The Coca-Cola-immersed sample had a highly irregular surface with a substantial amount of surface deposits. Regarding Omnichroma, the control sample exhibited a relatively smooth surface with minimal defects, indicating an intact and uniform composite structure under neutral conditions. The sample immersed in coffee showed increased surface degradation with small pits and cracks, suggesting degradation initiated by coffee components. The wine-immersed sample displayed moderate surface irregularities and minor erosions, indicating mild interaction between the wine and the composite surface (Figure 5).

### 3.5. Color Metrics and the Impact of Various Substances (Red Wine, Black Coffee, and Coca-Cola)

**Figure 6:**
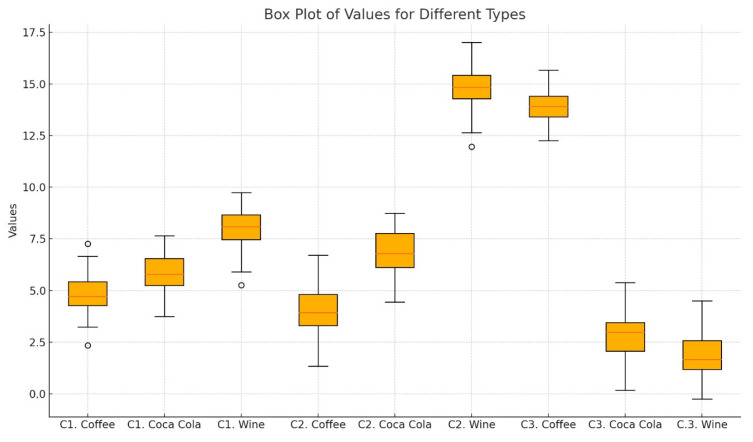
Descriptive statistics for sample groups by 10th day immersion (C1-Herculite Ultra XRV, C2 G-ænial A’chord, C3-Omnichroma).

The results indicate that Herculite UltraXRV experienced a moderate color change in all three substances, with ∆E values of 4.82 (±1.30) for coffee, 5.95 (±0.34) for red wine, and 3.99 (±0.52) for Coca-Cola. G-ænial A’CHORD showed the highest color change in coffee and red wine with ∆E values of 15.28 (±0.74) and 12.51 (±0.38), respectively, but a lower change in Coca-Cola with a ∆E of 2.79 (±0.16). Omnichroma had a high color change in coffee (∆E = 10.84 ± 1.03), a moderate change in red wine (∆E = 5.33 ± 0.27), and the lowest color change in Coca-Cola (∆E = 1.28 ± 0.44), as shown in Table 4 and Figure 6.

When considering the total color difference (ΔE), significant differences were observed after 10 days of immersion in the staining solutions. The ΔE value for Herculite Ultra XRV was significantly higher on Day 10, indicating a substantial color change (t (14) = 12.19, *p* < 0.001, Cohen’s d = 8.07). In case of G-ænial A’CHORD, the ΔE value increased significantly, showing a marked color change (t (14) = 18.86, *p* < 0.001, Cohen’s d = 21.11). For Omnichroma, the ΔE value also increased significantly, indicating a notable color change (t (14) = 17.28, *p* < 0.001, Cohen’s d = 16.98).

### 3.6. pH Values

The pH measurements of the staining solutions were as follows: Coca-Cola had a pH of 2.4, coffee had a pH of 5.6, and red wine had a pH of 3.5. These values indicate that the solutions range from highly acidic to mildly acidic.

## 4. Discussion

The surface quality of composite resin stands as a pivotal characteristic intricately tied to clinical triumph. Our findings align with those of previous studies, emphasizing that a smooth and impeccably polished composite resin surface significantly enhances esthetics, facilitates oral hygiene, and increases patient comfort, all of which contribute to higher clinical success rates. In contrast, rough surfaces present numerous challenges, such as discoloration, gingival irritation, increased plaque accumulation, and a greater risk of secondary caries and fractures. Polishing timing can significantly modify the surface characteristics of resin composites, influencing both their mechanical properties and esthetic outcomes. It has been well-documented that the duration and sequence of polishing steps can affect the surface smoothness and gloss of the composite material, which, in turn, impacts its resistance to staining and bacterial adhesion. For instance, a prolonged polishing duration can result in a smoother surface, which may reduce surface roughness and enhance the material’s resistance to discoloration and plaque accumulation. Conversely, insufficient polishing may leave the surface rough, increasing the likelihood of staining and microbial colonization. Therefore, the timing and thoroughness of the polishing process are critical factors that must be carefully controlled to ensure the optimal performance and longevity of the resin composite restorations. These observations highlight the essential role of surface smoothness and polish in ensuring the long-term efficacy and durability of composite resin restorations. Our study corroborates the existing literature, reinforcing the notion that optimizing surface quality is paramount for the clinical success of restorative dental materials [35,36].

Composite materials come with a diverse range of filler types, each influencing both their handling characteristics and physical properties. These advanced fillers increase the overall filler load, improve mechanical properties, and result in highly polishable surfaces [11]. Hybrid composites like Herculite XRV contain larger particle sizes compared to nanocomposites. This results in higher surface roughness after finishing and polishing, which increases the risk of surface discoloration [37].

The accurate characterization of material properties in studies like these holds paramount importance. Among the myriad surface properties, factors such as surface roughness, charge, and hydrophobicity assume critical roles in governing the bacterial adhesion process [12,38]. The hydrophobicity of the studied materials varied significantly. These results provide valuable insights into the hydrophobic properties of different resin composites, influencing their suitability for various applications. A comparison of our study results with those of previous research highlights distinct differences in the hydrophobicity and stain resistance of the studied materials. In the previous study, nanohybrid composite surfaces were hydrophilic, exhibiting a contact angle close to 80°, similar to the micro-hybrid composite surface with a contact angle of 86°. All materials had negative zeta potentials, ranging between 20.9 ± 0.8 mV and 22.2 ± 2.5 Mv [12]. In contrast, in our study, the nanocomposite Herculite Ultra, with its higher contact angle of 79.46°, demonstrated superior resistance to liquid absorption and, consequently, better stain resistance. In contrast, the micro-hybrid composite Omnichroma, with the lowest contact angle of 64.94°, was more prone to stain absorption due to its more hydrophilic nature. The hybrid composite G-Aenial A’CHORD, with a contact angle of 73.22°, showed moderate performance in terms of stain resistance, falling between Herculite Ultra and Omnichroma. Measuring the contact angle also enables the calculation of surface free energy (SFE), which provides insights into the polar or nonpolar interactions at the liquid/solid interface and reveals the hydrophilic or hydrophobic nature of a surface [13].

In our study, despite the observed differences in streaming potential, all materials exhibited negative charges, with streaming potential values ranging from approximately −30 mV to −50 mV. Specifically, Omnichroma showed the most negative streaming potential, indicating a stronger negative charge compared to G-Aenial and Herculite. These variations suggest differences in the surface charge density of the composite materials, which could influence their interaction with staining solutions and other substances. The clinical significance of the negative charge of dental resin composites lies in its influence on bacterial adhesion and biofilm formation. Negative surface charge on dental materials can repel negatively charged bacterial cells, thereby reducing bacterial colonization and subsequent biofilm formation. This property is particularly crucial in preventing secondary caries and periodontal diseases, which are often associated with bacterial biofilms on dental restorations. As discussed by Fugolin and Pfeifer (2017) [39], negatively charged surfaces, highly hydrophobic or hydrophilic surfaces, and nanoscale surface roughness have all been investigated as means to reduce bacterial attachment. Since bacterial cells are negatively charged, a normally negatively charged surface would repel bacteria and limit adhesion. However, it is important to note that some bacteria are remarkably resourceful and can adapt to better attach to negatively charged surfaces as well (Song et al., 2015) [40].

Bollen et al. proposed a threshold surface roughness (Ra = 0.2 μm) for bacterial retention, indicating that below this threshold, no significant reduction in bacterial accumulation is likely [41]. Polishing can reduce surface roughness to below the critical threshold. In our study, all specimens were polished to closely replicate clinical conditions, likely lowering the surface roughness below the specified threshold. The correlation between surface roughness (SR) and bacterial adhesion has been extensively studied, yielding varied results. While our research does not focus on bacterial adhesion, understanding the principles behind SR is crucial for optimizing restorative material surfaces. For instance, Hahnel et al. (2017) [42] found that for resin materials with SR greater than 0.2 μm, there was no significant correlation between bacterial adhesion and SR. Conversely, Bilgili et al. (2020) [43] emphasized that SR at or below the threshold level does not significantly affect bacterial adhesion. Another study from 2016 by Yuan et al. [44] indicated that rougher composite surfaces generally promote more bacterial adhesion. However, SR is primarily influential during the initial biofilm formation stages, with minimal effect on the biofilm’s final development. Yuan et al. (2016) also noted that surface free energy (SFE) might be more critical than SR for bacterial adhesion when SR values are very low (Ra ≤ 0.06 μm). These findings suggest a complex interaction between surface characteristics and bacterial behavior, necessitating further research to fully understand these dynamics and optimize dental material surfaces accordingly. These findings underscore the importance of surface characteristics in restorative materials. Although our study focuses on surface roughness, contact angle, streaming potential, and color changes, further research could explore the implications of SR and SFE on bacterial adhesion, potentially enhancing the performance and longevity of dental materials.

The acidic nature of Coca-Cola (pH 2.4) and red wine (pH 3.5) suggests a greater potential for the demineralization and surface degradation of the composite resins compared to coffee (pH 5.6). This process is similar to the demineralization of tooth enamel, where acidic substances erode the mineral content, leading to surface roughness and structural weakening [45]. This could explain the significant increase in surface roughness and color change observed in the composites after immersion in these solutions, highlighting the importance of selecting materials with enhanced resistance to acidic environments for long-term dental restorations.

The challenges surrounding esthetic restorations primarily stem from the intricacies of color matching procedures. Composites offer remarkable versatility, capable of being tailored to various shades and opacities that mimic natural dental structures’ optical properties [28].

In today’s age of highly esthetic dentistry, patients rightfully demand restorations that not only satisfy esthetic standards but also maintain their initial color and appearance over an extended period. Hence, achieving proper color matching with the surrounding tooth tissue holds paramount importance not only initially but also for the restoration’s long-term durability. Numerous studies have investigated the effects of resin materials on discoloration after being immersed in various beverages. These studies have examined the coloring effects of a wide range of substances, including tea, coffee, cola, wine, soy sauce, grape juice, chlorhexidine, vinegar, ayran, orange juice, and yogurt, on composite resins. In comparison to these studies, in our study, the composites were immersed for 20 min daily over a period of 10 consecutive days. The water absorption and solubility of composite resins are influenced by the duration of exposure. Studies involving tea, coffee, wine, and cola have varied application times. For example, Villalta et al. (2006) immersed samples in color solutions for 3 h daily for 40 days, followed by 21 h in distilled water. Bagheri et al. (2005) kept samples in colorant solutions for 1 week, then in distilled water for another week. Dietschi conducted the staining experiment over 3 weeks. Çelik et al. (2016) kept samples in coffee, red wine, cola, and distilled water for 3 h daily, measuring the color with a spectrophotometer on the 1st, 7th, 15th, and 30th days [46]. In the study conducted by Barutcigil and Yildiz which aimed to evaluate external color changes in dimethacrylate- and silorane-based composites, five different composite materials were immersed in red wine, coffee, cola, tea, and distilled water, followed by color measurements. The results showed that red wine caused the most significant color change in all resins. It was reported that all restorative materials were susceptible to discoloration from these commonly consumed beverages, particularly wine and coffee [47].

The variations in color parameters following immersion in red wine, black coffee, and Coca-Cola suggest that these substances adhere to the composite surfaces to varying degrees. Coca-Cola, with its highly acidic pH, likely contributes to a significant alteration in color, similar to the pronounced color changes observed with black coffee and red wine. The higher ΔE value for black coffee compared to red wine underscores the more pronounced alteration in color, further emphasizing the influence of the staining solution’s composition and pH on the composite materials.

Aesthetic restorative materials must exhibit outstanding physical and mechanical properties, as well as color stability. Guler et al. noted that the consumption of coffee, tea, and wine and smoking results in extrinsic color changes in these materials. Dietschi et al. pointed out that physiochemical stress leads to the surface disintegration of restorative materials, increasing their susceptibility to color change. Moreover, frequent consumption of low pH beverages has been demonstrated to erode teeth. Given the increase in acidic beverage consumption and the rising demand for esthetic restorative materials, continuous advancements are being made in this field [20].

In our study, a comparative analysis was performed to assess the changes in surface roughness (SR) and color stability (ΔE) for each composite resin after 10 days of immersion in staining solutions. For Herculite UltraXRV, the results indicate that while color changes were notable, roughness changes were less pronounced, with red wine causing the highest roughness increase. G-ænial A’CHORD exhibited significant color changes, especially in coffee and red wine, while Coca-Cola caused the highest increase in roughness. Omnichroma showed significant color changes, particularly in coffee, with moderate changes in roughness. Awliya et al. stated that there was no significant difference in the microhardness of resin-based composite materials before and after immersion in coffee. Badra et al. found that the microhardness of materials immersed in coffee and Coca-Cola remained stable for up to 7 days but decreased after 30 days [20].

The adherence of food and beverages to dental surfaces significantly influences the adhesion and proliferation of microorganisms. Therefore, compounds exhibiting antimicrobial properties are of particular interest in dental research. Phenolic compounds, abundant in red wine and black coffee, have drawn attention due to their potential antimicrobial effects. Considering the widespread consumption of these beverages, their impact on dental materials is a crucial area of investigation.

This study found that beverages significantly affect the surface characteristics of dental materials, including hydrophobicity, surface roughness, and color stability. Herculite Ultra XRV had the highest hydrophobicity, making it more resistant to stains, while Omnichroma had the lowest, making it more prone to staining. G-ænial A’CHORD had moderate hydrophobicity. After 10 days in acidic beverages, surface roughness increased for all materials, with G-ænial A’CHORD showing the most roughness in Coca-Cola and Herculite Ultra XRV in red wine. Omnichroma showed moderate roughness changes in coffee. In terms of color stability, G-ænial A’CHORD had the most significant changes, especially in coffee and red wine; Omnichroma showed substantial changes in coffee but least in Coca-Cola, and Herculite Ultra XRV had moderate changes across all beverages.

The findings of this study must be considered within the context of its limitations. This study was conducted in vitro, which does not fully replicate the complex conditions of the oral environment, such as varying pH levels, temperature fluctuations, duration of exposure to the beverages, and mechanical stresses from chewing and brushing. Additionally, the immersion period was limited to 10 days, which may not capture the long-term effects of these beverages on the materials. Also, one of the limitations of our study is the timing and thoroughness of polishing which can significantly influence the surface characteristics of resin composites. Extended polishing can produce smoother surfaces with enhanced resistance to staining and bacterial adhesion, while insufficient polishing may leave the surface rough, increasing susceptibility to discoloration and microbial colonization. The sample size was also limited, potentially affecting the generalizability of the findings. Furthermore, only three types of resin composites were evaluated, which may not represent the full spectrum of available materials. Future studies should consider longer immersion periods, different polishing times and techniques, larger sample sizes, and a broader range of composite materials to better understand their influence on the surface properties and overall performance of dental resin composites. Incorporating real-life variables such as saliva, plaque, and food particles would also provide a more comprehensive understanding of the interactions between dental materials and the oral environment.

## 5. Conclusions

This study highlights the significant impact of surface characteristics on the performance and longevity of dental restorative materials. The analysis of three commercial dental resin composites demonstrated that exposure to acidic beverages like red wine, black coffee, and Coca-Cola leads to notable changes in surface roughness and color stability. The nanohybrid composite, with the highest contact angle, exhibited superior hydrophobicity and resistance to staining compared to the more hydrophilic hybrid resin composite. These findings underscore the importance of selecting materials with enhanced resistance to acidic environments to minimize degradation and maintain esthetic and functional integrity. Furthermore, antioxidant-rich beverages such as black coffee and red wine can influence surface properties, potentially affecting color stability, with black coffee causing more substantial alterations. Future research should focus on incorporating antibacterial agents into dental composites to mitigate bacterial adhesion and enhance restoration longevity. Additionally, investigating the influence of different foods on surface properties and employing more realistic in vitro models that mimic oral conditions can provide deeper insights into material performance and guide the development of superior restorative solutions.

## Figures and Tables

**Table 1:** Specifications of the materials used in the study.

Product Name	Type	Resin Matrix	Filler Content	Color	Lot Number	Manufacturer
Herculite Ultra XRV [16]	Nanohybrid resin composite	Bis-GMA, TEGDMA	78% wt, 59% vol	A2	10198512	Kerr, Italy
G-Aenial A’CHORD [17]	Hybrid resin composite	Bis-MEPP-based resin	82% wt, 56% vol	Bleach	230328C	Gc Tokyo, Japan
Omnichroma [18]	Supra-nanospherical resin composite	UDMA, TEGDMA	79% wt, 68% vol	Universal	123E83	Tokuyama-Dental, Japan

**Table 2:** Mean contact angle and standard deviation of the materials used in the study.

Resin Composite	Mean Contact Angle	Standard Deviation
Herculite Ultra	79.46	6.52
G-ænial A’CHORD	73.22	2.71
Omnichroma	64.94	3.08

**Table 3:** The surface roughness (SR) and standard deviation—initial and after 10 days’ immersion.

Type of Resin Composite	Initial SR (SD)	10 Days Coffee Immersion (SD)	Paired *t*-Test	10 Days Red Wine Immersion (SD)	Paired *t*-Test	10 Days Coca-Cola Immersion (SD)	Paired *t*-Test
Herculite UltraXRV	1.66 (±0.45)	1.36 (±0.23)	t (14) = −4.28, *p* < 0.001	2.28 (±0.77)	t (14) = 3.42, *p* < 0.01	1.63 (±0.26)	t (14) = 1.75, *p* = 0.098
G-ænial A’CHORD	1.84 (±0.33)	2.33 (±0.62)	t (14) = 4.12, *p* < 0.001	1.52 (±0.63)	t (14) = −2.31, *p* = 0.037	3.98 (±0.13)	t (14) = 9.53, *p* < 0.001
Omnichroma	1.66 (±0.09)	1.53 (±0.38)	t (14) = 4.15, *p* < 0.001	1.15 (±0.08)	t (14) = 2.07, *p* = 0.055	1.21 (±0.31)	t (14) = 2.28, *p* = 0.039

**Table 4:** Color parameters (∆E) for materials immersed in red wine, black coffee, and Coca-Cola.

	∆E 10 Days Coffee Immersion	∆E 10 Days Red Wine Immersion	∆E 10 Days Coca-Cola Immersion
Herculite UltraXRV	4.82 (±1.30)	5.95 (±0.34)	3.99 (±0.52)
G-ænial A’CHORD	15.28 (±0.74)	12.51 (±0.38)	2.79 (±0.16)
Omnichroma	10.84 (±1.03)	5.33 (±0.27)	1.28 (±0.44)

## Data Availability

The data presented in this study are available on request from the corresponding author.
